# EFFECTIVENESS OF A COMMUNITY HEALTH WORKER-BASED INTERVENTION TO IMPROVE DIETARY HABITS: A CROSSOVER TRIAL IN JAPAN

**DOI:** 10.1093/geroni/igz038.504

**Published:** 2019-11-08

**Authors:** Hiroshi Murayama, Atsuko Taguchi, Takuhiro Yamaguchi

**Affiliations:** 1 Institute of Gerontology, The University of Tokyo, Tokyo, Japan; 2 Department of Public Health Nursing, Tohoku University Graduate School of Medicine, Sendai, Miyagi, Japan; 3 Division of Biostatistics, Tohoku University Graduate School of Medicine, Sendai, Miyagi, Japan

## Abstract

Background: Community health workers (CHWs), often called “health promotion volunteers” in Japan, are individuals who act as a natural helping resource in the community. This study tested the effectiveness of a CHW-based intervention in improving dietary habits among community-dwelling older people in Japan, using a controlled, cross-over design. Methods: Seventy-eight people aged 65–74 years with poor dietary variety living in four districts in Hikone City (Shiga Prefecture, Japan) were allocated to an immediate-intervened group (IIG; n = 41) or a delayed-intervened group (DIG; n = 37). Participants joined a bi-weekly, four-session program (120 minutes/session), comprising “CHWs’ drama-style lectures,” “group discussion among participants and CHWs,” “tasting of dishes,” and “take-home practical activities.” For the initial 2-month period, the IIG received the intervention and the DIG did not. The groups were crossed over for the subsequent 2-month period. The primary outcome measure was the participants’ dietary variety scale score. Results: The dietary variety scale score in the IIG significantly increased in the initial 2-month period compared with the DIG (effect size 1.60 points; 95% confidence interval 0.75, 2.45). The intervention had a similar effect in the DIG in the subsequent 2-month period. Moreover, an analysis within the IIG showed that the intervention effects persisted for at least 2 months after the intervention. Conclusions: The CHW-based intervention improved dietary habits among older people. Our findings provide evidence that a CHW-based natural helping approach is a possible solution to promote healthy aging in the community.

